# Hypertension Secondary to PHPT: Cause or Coincidence?

**DOI:** 10.1155/2011/974647

**Published:** 2011-03-07

**Authors:** Helmut Schiffl, Susanne M. Lang

**Affiliations:** ^1^Department of Internal Medicine-Campus Innenstadt, University Hospital, University of Munich, 80336 Munich, Germany; ^2^KfH Nierenzentrum München-Laim, Elsenheimerstra*β*e 63, 80687 München, Germany; ^3^SRH Wald-Klinikum Gera, 2. Medizinische Klinik, 07548 Gera, Germany

## Abstract

Primary hyperparathyroidism (PHPT) may be associated with arterial hypertension. The underlying mechanisms are not fully understood and reversibility by parathyroid surgery is controversial. This study aimed to characterize pressor hormones, vascular reactivity to norepinephrine, and cytosolic-free calcium in platelets in 15 hypertensive patients with hypercalcaemic PHPT before and after successful parathyroidectomy and to compare them with 5 pre-hypertensive patients with normocalcaemic PHPT, 8 normotensive patients with hypercalcaemic PHPT and 15 normal controls. Hypertensive patients with hypercalcaemic PHPT had slightly higher levels of pressor hormones (*P* < 0.05), enhanced cardiovascular reactivity to norepinephrine (*P* < 0.05) and increased cytosolic calcium in platelets (*P* < 0.05) than controls. Pre-hypertensive patients with normocalcaemic PHPT had intermediate values of increased cardiovascular reactivity and cytosolic calcium. Normotensive patients with hypercalcaemic PHPT and normotensive controls had comparable pressor hormone concentrations and intracellular calcium levels. Successful parathyroidectomy was associated with normal blood pressure values and normalisation of pressor hormone concentrations, cardiovascular pressor reactivity and cytosolic free calcium. Our results suggest that parathyroid hypertension is mediated/maintained, at least in part, by functional alterations of vascular smooth muscle cells and can be cured by parathyroidectomy in those patients who do not have primary hypertension.

## 1. Introduction

Sporadic primary hyperparathyroidism (PHPT) is an endocrine disorder usually characterized by persistent fasting hypercalcaemia attributable to autonomous overproduction of parathyroid hormone (PTH) by parathyroid adenoma or hyperplasia (hypercalcaemic PHPT). However, a proportion of patients with PHPT (20%) show normal total and ionized serum calcium levels in the presence of persistently elevated PTH concentrations [1–3]. Initially, there have been questions about the existence of this new clinical phenotype of PHPT, as well as about the correctness of the term “normocalcaemic PHPT”. However, at the 2002 consensus conference, normocalcaemic PHPT was appreciated as a distinct entity of PHPT. Whether or not normocalcaemic PHPT represents an earlier form or a forme fruste of the disease is subject to discussion [4]. 

PHPT is associated with increased risk of arterial hypertension. Recent investigations have reported high blood pressure in 40 to 65% of patients with PHPT [5–9]. Despite variations in published data due to different patient selection criteria, the prevalence of hypertension in patients with PHPT is higher than in the general population regardless of age [5, 10]. However, elevated PTH levels have also been reported in a subgroup of patients with primary (essential) hypertension [11]. Thus, in daily practice the association of high concentrations of PTH with high blood pressure may not necessarily have a causal relationship as PHPT and primary hypertension are frequent and may coincide in patients older than 50 years. Consistent with the assumption of coincidence of both diseases is the observation that more than 50% of PHPT patients remain hypertensive in spite of successful parathyroidectomy.

At present the mechanisms underlying parathyroid hypertension are not clear. Systemic hypertension accompanying hypercalcaemic PHPT is haemodynamically characterized by increased total peripheral vascular resistance [12, 13]. Proposed factors producing hypertension in PHPT include (a) abnormalities in major endocrine pressor factors, such as the sympathetic nervous system and/or the renin-angiotensin-aldosterone axis [14, 15] and (b) dysfunction or structural changes of resistance vessels documented either by an altered vasodilatory response [16, 17] and/or an enhanced vascular constriction to pressor hormones [18–20].

Regardless of the precise nature of the operating factors, abnormal calcium metabolism at the level of the vascular smooth muscle cells may be the final common pathway for functional alterations of vascular tone and vascular reactivity and finally for increased total peripheral vascular resistance.

The aim of this study was to evaluate circulating levels of vasopressor hormones, cytosolic calcium in platelets and cardiovascular pressor response to norepinephrine in patients with different forms of PHPT and to characterize the changes in blood pressure and in proposed causes of blood pressure dysregulation in patients with hypercalcaemic PHPT and hypertension before and after parathyroidectomy. 

## 2. Patients and Methods

### 2.1. Study Population

Three groups of patients and one group of healthy subjects were included in the study. Group 1 were fifteen patients with hypercalcaemic PHPT and untreated chronic arterial hypertension (RR ≥ 140/90 mm Hg). Group 2 included 8 patients with hypercalcaemic PHPT and normotension (blood pressure less than 120/80 mm Hg). Group 3 included 5 patients with normocalcaemic PHPT and untreated prehypertension (RR 120–139 mm Hg/80–89 mm Hg), and Group 4 consisted of 15 healthy normotensive subjects (age-, sex-, and body weight-matched to patients from Group 1). Patients with PHPT were accepted only if they had no family history of primary hypertension or known long-standing personal history of primary hypertension. The known duration of arterial hypertension was short in all patients (less than 6 months). Antihypertensive drugs were discontinued at least 4 weeks before the study. None of the participants of the investigation had taken any medication interfering with platelet function or production for at least 3 weeks. Informed written consent was obtained from all study participants, and the study protocol was approved by the local ethics committee [21]. 

#### 2.1.1. Characteristics of the Study Groups (Table 1)

The three patient groups and the normal subjects did not differ in mean age, in the predominance of female participants, and had similar renal function. The two patient groups (Group 1 and 2) with hypercalcaemic PHPT did not differ in mean serum total calcium concentrations, mean serum phosphate concentrations, and mean iPTH levels. Compared to these two hypercalcaemic PHPT patient groups, the normocalcaemic patients from Group 3 had significantly lower PTH levels. The prevalence of left ventricular hypertrophy (LVH) was high in all patients affected by PHPT (10 out of 15 patients from Group 1, 5 out of 8 patients from Group 2 and 3 out of 5 patients from Group 3). Neither the prevalence of LVH nor its severity showed a correlation with high blood pressure. 

#### 2.1.2. Definitions and Classifications

**(a) Sporadic PHPT**

Hypercalcaemic PHPT was defined by persistent supranormal total serum calcium levels (more than 2.6 mmol/L) and supranormal ionized calcium (more than 1.35 mmol/L) together with supranormal serum intact PTH (iPTH) level (more than 55 ng/L). Normocalcaemic PHPT was defined by the repeated demonstrations of consistently normal serum total and ionized calcium levels with persistently abnormal serum iPTH. The diagnosis of normocalcaemic PHPT was made when vitamin-D deficiency by measurements of 25-hydroxy vitamin D3 (less than 20 ng/mL) was excluded. None of these patients had any family history of hypercalcaemia; they all had normal or high urinary calcium excretion to an extent excluding familial hypocalcuric hypercalcaemia. None of the patients with normo- or hypercalcaemic PHPT had reduced glomerular filtration (estimated by creatinine clearance) or was taking thiazides, lithium or other drugs known to affect calcium metabolism.

**(b) Chronic Arterial Hypertension**

Chronic arterial hypertension was defined by constant blood pressure values above 140/90 mm Hg documented on repeated readings at clinical visits or at home. Normotension was defined as blood pressure values below 120/80 mm Hg. The Joint National Committee on Prevention, Detection, Evaluation, and Treatment of high blood pressure, defined prehypertension as a predisease state with systolic blood pressures ranging from 120 to 139 mm Hg and diastolic blood pressures from 80 to 89 mm Hg [22]. The classification of blood pressure as normal, pre-hypertensive or hypertensive was confirmed by 24-hour ambulatory blood pressure monitoring.

In contrast to primary (essential) hypertension, secondary hypertension was defined as high blood pressure which is caused by an identifiable underlying cause (renal, haematologic, or cardiovascular diseases, endocrine disorders, or drug use). Secondary forms of high blood pressure other than parathyroid hypertension were excluded by clinical examination and established tests including estimation of renal function, urinalysis, renal ultrasound, Duplex and Doppler ultrasound, determinations of haematocrit, serum electrolytes and fasting blood glucose, hormonal analysis of thyroid, and if appropriate of adrenal function and chest radiograph.

**(c) Left Ventricular Hypertrophy**

Echocardiography was performed using two-dimensional guided M-mode echocardiography according to the recommendations of the American Society of Echocardiography [23]. Left ventricular mass was estimated with the anatomically validated formula by Devereux et al. [24] and defined as LV mass index 49.2 g/m² in men and 46.7 g/m² in women.

### 2.2. Study Protocol

Blood pressure recordings were performed using a standard cuff and sphygmomanometer, and blood samples were taken through an intravenous cannula for the determination of serum total calcium and inorganic phosphate (by autoanalyzer techniques), iPTH (by a two site immunoreactive assay for 1–84 human parathyroid hormone monoclonal antibodies), plasma renin activityand plasma aldosterone (by radioimmunoassay), and plasma norepinephrine (by radioenzymatic method). Intracellular free calcium concentrations in platelets were measured by Quin-2AM [25].

The cardiovascular pressor reactivity to intravenously infused norepinephrine (1-norepinephrine tartrate in 5% glucose) was determined while the participants were in the supine position as described previously [21]. The rate of infused norepinephrine was increased stepwise until the blood pressure stabilized for at least 10 minutes at different levels. Two targets, namely, 10 to 15 mm Hg and 25 to 30 mm Hg above the basal mean arterial blood pressure, were chosen. The pressor dose of norepinephrine was defined as the dose necessary to increase the mean blood pressure by 20 mm Hg.

The cardiovascular pressor response to norepinephrine was studied in hypercalcaemic PHPT patients with hypertension prior to surgery and 3 months after surgical correction of PHPT. Normocalcaemic Patients with PHPT and normotensive controls were studied once. 

Study Group 1 was tested twice, that is, prior to surgery and after 3 months after surgery; Groups 3 and 4 were studied once, and Group 2 was not available for study.

Hypercalcaemic PHPT with normotension were not available for this part of the investigations. 

### 2.3. Data Analysis

Results are given as means ± SD. Natural logarithmic transformation of plasma renin activity, plasma aldosterone, plasma norepinephrine and pressor dose was done, and comparisons between groups were performed using the Mann-Whitney *U* test. The Student paired test was used for comparison of the pre- and postoperative values in the hypertensive hypercalcaemic PHPT patients. Correlations were calculated by the Spearman rank method. *P* values less than 0.05 were regarded as significant. All statistical calculations were done with the SPSS version 10 (SPSS Inc., Chicago, Il, USA). 

## 3. Results

Blood pressure, pressor hormones, cardiovascular norepinephrine reactivity, and cytosolic calcium in platelets in the study groups are given in Table 2.

Systolic, diastolic, and mean blood pressure levels in hypercalcaemic PHPT patients with hypertension (Group 1) were significantly higher than in hypercalcaemic PHPT patients with normotension (Group 2) or in healthy subjects (Group 4). Prehypertensive patients with normocalcaemic PHPT (Group 3) had intermediate values. 

Patients from Group 1 had significantly higher mean plasma renin activity, plasma aldosterone, and norepinephrine levels than matched healthy subjects from Group 4. There were no significant differences between healthy subjects and patients from Group 2 and 3. None of these pressor hormones correlated positively either with blood pressure or PTH levels. 

The pressor doses of norepinephrine were tested in Group 1, 3, and 4. They were significantly lower in Group 1 (hypertensive patients with hypercalcaemic PHPT) and in Group 3 (prehypertensive patients with normocalcaemic PHPT) by comparison with healthy subjects from Group 4. 

There were significant differences in mean platelet calcium concentrations between Group 1 and Group 3, respectively, compared to patients from Group 2 and Group 4. A close correlation between platelet cytosolic calcium levels and mean blood pressure was apparent when all 43 participants were included (*r* = 0.9, *P* < .01).

Preoperative values of all patients from Group 1 are given in Tables 1 and 2. Removal of a parathyroid adenoma (13 cases) or subtotal (3.5) parathyroidectomy (2 cases of parathyroid hyperplasia) was successful in all hypertensive patients with hypercalcaemic PHPT. Circulating parathormone concentrations (32 ± 5 pg/mL) and serum calcium levels (2.3 ± 0.1 mmol/L) were normalized in all patients. Three months after surgery, blood pressure recordings documented a reduction of high blood pressure towards normotension in all patients (130/78 ± 6/4 mmHg). Norepinephrine pressor doses (135 ± 16 ng/kg/min) were higher and plasma renin activity (1.3 ± 0.3 ng/mL/hr), plasma aldosterone concentrations (4.1 ± 1.3 ng/100 mL), and plasma norepinephrine levels (23 ± 3 ng/100 mL) were significantly lower after parathyroidectomy and did not differ from values obtained from control subjects. Mean cytosolic calcium levels in successfully treated hypercalcaemic PHPT patients were significantly lower than prior surgery (105 ± 6 nM/L) and comparable to values in normotensive subjects. 

## 4. Discussion

The main results of the present investigations demonstrate that short-standing (known duration of high blood pressure less than 6 months) parathyroid hypertension can be cured by normalization of the hyperactive parathyroid metabolism in patients who do not have primary hypertension.

The pathophysiology of hyperparathyroid hypertension is still an area of research, but at least three theories have been proposed to explain increased total peripheral resistance [12, 13]. Firstly, major pressor factors causing vasoconstriction such as the renin-angiotensin-aldosterone system and the activity of the peripheral sympathetic nervous system were found to be higher in hypertensive patients with PHPT compared to controls. These abnormalities were reversed by successful parathyroidectomy. However, the differences in these pressor factors among hypertensive patients with PHPT and age-, sex- and body weight-matched controls were small. Nevertheless, the pressor effect of any vasoactive factor depends not only on its circulating concentration but also on its cardiovascular reactivity.

Secondly, one major finding of our present study is the demonstration of a significantly enhanced pressor response of the cardiovascular system to norepinephrine, although circulating plasma norepinephrine levels were slightly increased or normal in all patients with PHPT. These data are in line with other observations [14, 15, 26] and they suggest that parathyroid hypertension may be initiated and maintained by an exaggerated cardiovascular response to vasoactive hormones. The enhanced cardiovascular reactivity to norepinephrine could be the consequence of functional alterations or structural changes in the blood vessel walls.

The concentration of intracellular calcium in platelets has been found to be elevated in primary and various forms of secondary hypertension [25, 27–32]. We and other investigators measured highly increased cytosolic free calcium levels in platelets in hypercalcaemic hypertensive patients with PHPT, intermediately increased cytosolic free calcium levels in prehypertensive patients with normocalcaemic PHPT [33] and normal levels in normotensive hypercalcaemic patients with PHPT [26, 34]. It has to be kept in mind, however, that these findings in platelets cannot necessarily be extrapolated to cytosolic levels in vascular muscle cells. However, there was a strong correlation between platelet cytosolic calcium concentrations and mean arterial pressure when all groups of participants were considered jointly. The elevated intracellular calcium concentrations found in our patients with PHPT were not irreversible, since they could be reverted to the normal range after successful parathyroidectomy.

If the results obtained in platelets are indeed representative for vascular smooth muscle cells, functional alterations characterized by an abnormal calcium metabolism at the level of vascular smooth muscle cells may be the final common pathway for the enhanced vascular reactivity to pressor hormones and the increased vascular tone and finally for increased peripheral vascular resistance. 

Thirdly, structural changes in the vessels may maintain high blood pressure in PHPT, but such changes may occur as a result of either longstanding hypertension, hypercalcaemia, or metabolic abnormalities associated with PHPT (dyslipidaemia, diabetes), or a combination of these. Structural changes can lead to impaired vasoreactivity in patients with PHPT which is not improved by successful parathyroidectomy [18]. Rodriguez-Portales and Fardella [19] found a persisting abnormal pressor reactivity to norepinephrine in 7 normotensive patients with PHPT after parathyroidectomy, and these authors postulated secondary structural changes in the vascular walls. To what extent structural changes in the resistance vessel contribute to the maintenance of hypertension in patients with PHPT is largely unknown.

The factors underlying the disturbances in cellular calcium metabolism found in hypertension due to PHPT are not well defined. There are objections against the hypothesis that hypercalcaemia per se or PTH causes enhanced cellular influx of calcium and raised cytosolic calcium. In fact, hypercalcaemic PHPT may be associated often with normotension, questioning the role of excess calcium or increased PTH. Moreover, preincubation of platelets of normotensive patients with exogenous iPTH in vitro had no effect on baseline cytosolic calcium [26, 28, 35]. Lewanczuk et al. [36] showed that the diseased parathyroid glands in hypertensive patients with PHPT express a low molecular weight circulating parathyroid hypertensive factor (PHF) that was significantly higher in hypertensive patients with PHPT than in normotensive patients with PHPT. Postoperatively, PHF was undetectable in PHF-positive patients. Our previous in vitro studies with platelets obtained from normotensive controls and plasma derived from hypertensive patients with PHPT are well comparable with in vitro studies by Barbagallo et al. [37] who found that PHF increased free intracellular calcium in erythrocytes. Unfortunately the molecular structure of this putative factor is still not elucidated.

Left ventricular hypertrophy is a common finding in patients with PHPT although this finding may be related primarily to excess parathyroid hormone secretion and not to hypertension. Previous studies have shown that successful parathyroidectomy may lead to reversal of left ventricular hypertrophy even in PHPT patients with normal resting blood pressure [38]. 

## 5. Conclusion

Given the high prevalence of chronic arterial hypertension (30% of the US population [39]) and the relatively low prevalence of PHPT (0.1–0.7% [40, 41]), there is no doubt that PHPT may often coexist with primary chronic arterial hypertension. Both forms of hypertension are lacking a measurable biochemical factor differentiating hypertensive factors pathognomonic for one or the other. This coexistence may however explain the discrepant results of parathyroidectomy on high blood pressure in this patient group.

Undoubtedly, there is a need for further investigations to clarify the pathogenesis of parathyroid hypertension. 

## Figures and Tables

**Table 1:** Characteristics of patients with PHPT (Group 1–3) and age-and sex matched normal subjects (Group 4).

	Group 1	Group 2	Group 3	Group 4
*N*	15	8	5	15
age, years	58 ± 9	61 ± 5	60 ± 8	58 ± 12
Gender(F/m)	9/6	5/3	4/1	9/6
GFR, mL/min/1.73 m²	115 ± 9	120 ± 8	111 ± 8	121 ± 12
Serum calcium mMol/L	3.2 ± 0.2*	3.1 ± 0.1*	2.5 ± 0.1*	2.3 ± 0.1
Serum phosphate, mMol/L	0.8 ± 0.1*	0.8 ± 0.2*	1.0 ± 0.2*	1.4 ± 0.2
Serum PTH, pg/mL	212 ± 31*	292 ± 28*	97 ± 13*	32 ± 6

*P < 0.05 versus healthy subjects.

Group 1 hypercalcaemic, hypertensive patients with PHPT.

Group 2 hypercalcaemic, normotensive patients with PHPT.

Group 2 normocalcaemic, pre-hypertensive patients with PHPT.

Group 2 normal subjects.

**Table 2:** Blood pressure, pressor hormones, cytosolic calcium and cardiovascular reactivity to norepinephrine in PHPT patients and age and sex matched normotensive subjects.

	Group 1	Group 2	Group 3	Group 4
Blood pressure, mmHg	171/111 ± 10/8*	118/70 ± 4/3	135/85 ± 3/2	120/70 ± 4/3
Plasma Renin activity ng/mL/h	1.6 ± 0.2*	1.5 ± 0.1	1.3 ± 0.3	1.4 ± 0.2
Plasma aldosterone ng/100 mL	5.2 ± 1.1*	4.5 ± 0.9	4.7 ± 0.9	4.2 ± 1.1
Plasma norepinephrine ng/100 mL	28 ± 3*	24 ± 2	25 ± 3	24 ± 2
Platelet calcium nMol/L	161 ± 18*	105 ± 12	135 ± 12*	112 ± 15
Norepinephrine pressor dose ng/kg/min	95 ± 13*	Not done	115 ± 12*	132 ± 15

*P < 0.05 versus control subjects.

Group 1 hypercalcaemic, hypertensive patients with PHPT.

Group 2 hypercalcaemic, normotensive patients with PHPT.

Group 2 normocalcaemic, pre-hypertensive patients with PHPT.

Group 2 normal subjects.
